# Dietary Supplementation with Palmitoyl-Glucosamine Co-Micronized with Curcumin Relieves Osteoarthritis Pain and Benefits Joint Mobility

**DOI:** 10.3390/ani10101827

**Published:** 2020-10-08

**Authors:** Enrico Gugliandolo, Alessio Filippo Peritore, Daniela Impellizzeri, Marika Cordaro, Rosalba Siracusa, Roberta Fusco, Ramona D’Amico, Rosanna Di Paola, Carlo Schievano, Salvatore Cuzzocrea, Rosalia Crupi

**Affiliations:** 1 Department of Chemical, Biological, Pharmaceutical and Environmental Science, University of Messina, 98168 Messina, Italy; egugliandolo@unime.it (E.G.); aperitore@unime.it (A.F.P.); dimpellizzeri@unime.it (D.I.); cordarom@unime.it (M.C.); rsiracusa@unime.it (R.S.); rfusco@unime.it (R.F.); rdamico@unime.it (R.D.); dipaolar@unime.it (R.D.P.); 2 Innovative Statistical Research srl, Prato Della Valle 24, I-35123 Padova, Italy; cs@i-stat.it; 3 Department of Pharmacological and Physiological Science, Saint Louis University School of Medicine, Saint Louis, MO 63104, USA; 4 Department of Veterinary Science, University of Messina, 98168 Messina, Italy; rcrupi@unime.it

**Keywords:** osteoarthritis, mobility, pain, allodynia, hyperalgesia, inflammation, palmitoyl-glucosamine, curcumin, endocannabinoid system, ALIAmides

## Abstract

**Simple Summary:**

Canine osteoarthritis is a chronic degenerative joint disease and a major cause of elective euthanasia. The disorder increasingly limits joint motion, might cause lameness as well as pain, and impacts quality of life. An unmet need remains for safe and effective therapies for osteoarthritis. Palmitoyl-glucosamine and curcumin are used in animal nutrition. A co-micronized formulation, with the two substances being jointly processed to reduce their particle size and increase the extent to which they can be absorbed, is currently available on the European market. The present study investigated if this formulation could relieve joint pain and benefit mobility. Two well-established rat models of inflammation and osteoarthritis pain were used. Results from the first set of experiments showed that the dietary supplement relieved experimentally induced paw edema, infiltration of inflammatory cells, and decreased sensitivity to painful stimuli (thermal hyperalgesia). In the osteoarthritis model, the supplement proved to protect joint cartilage against degradation and successfully address neuropathic pain (i.e., pain from normally non-painful stimuli). Locomotor function recovered by 45% under supplement administration. The present findings suggest that the dietary supplement with palmitoyl-glucosamine co-micronized with curcumin might help manage osteoarthritis.

**Abstract:**

Chronic mixed pain and orthopedic dysfunction are the most frequently associated consequences of canine osteoarthritis (OA). An unmet need remains for safe and effective therapies for OA. Palmitoyl-glucosamine (PGA) and curcumin are safe and naturally occurring compounds whose use is limited by poor bioavailability. Micronization is an established technique to increase bioavailability. The aim of this study was to investigate if the dietary supplementation with PGA co-micronized with curcumin (PGA-Cur, 2:1 ratio by mass) could limit pathologic process in two well-established rat models of inflammation and OA pain, i.e., subplantar carrageenan (CAR) and knee injection of sodium monoiodoacetate (MIA), respectively. In CAR-injected animals, a single dose of PGA-cur significantly reduced paw edema and hyperalgesia, as well as tissue damage and neutrophil infiltration. The repeated administration of PGA-Cur three times per week for 21 days, starting the third day after MIA injection resulted in a significant anti-allodynic effect. Protection against cartilage damage and recovery of locomotor function by 45% were also recorded. Finally, PGA-cur significantly counteracted MIA-induced increase in serum levels of TNF-α, IL-1β, NGF, as well as metalloproteases 1, 3, and 9. All the effects of PGA-Cur were superior compared to the compounds used singly. PGA-Cur emerged as a useful dietary intervention for OA.

## 1. Introduction

Osteoarthritis (OA) is a common progressive joint disease in dogs and cats [1,2,3,4]. Chronic pain and orthopedic dysfunction are the most frequently associated consequences of canine and feline OA [5,6,7], with under-recognized and undermanaged chronic pain eventually resulting in premature euthanasia [7]. Once believed to be limited to the articular cartilage, OA is now considered a disease of the whole joint, initiated and maintained by a complex cross-talk between subchondral bone, cartilage, and synovial membrane [8]. It is currently viewed as a multifactorial disorder, with chondrodegeneration and low-grade chronic inflammation concurrently contributing to progressive cartilage loss, pain, and joint dysfunction [9]. Targeting just one of these processes could thus be insufficient to benefit OA patients. Most notably, OA pain is evoked by locally released mediators (e.g., amines, cytokines, and nerve growth factor) which can directly activate the nociceptors or modulate their sensitivity, thus causing a hyperreactive reaction to stimuli. Normal weight bearing in the presence of mechanical deformation of the joint may also activate high-threshold synovial mechanoreceptors [10]. As a result, normal stimuli are perceived as painful (allodynia) and normally painful stimuli cause pain of greater intensity (hyperalgesia). Neuroplastic changes contribute to hyperalgesia and allodynia, the main characteristics of OA pathological pain [11]. Such neuroplastic changes can occur in primary afferent terminals (peripheral sensitization) as well as in the spinal cord and brain (central sensitization) [10,11,12] and, respectively, involve peripheral immune cells (e.g., mast cells) and spinal microglia [8,10,13].

Despite being a first-choice therapy, non-steroidal anti-inflammatory drugs (NSAIDs) are symptom-modifying agents and have little benefit on the neuro-immune changes sustaining OA pain [9,14]. Moreover, NSAIDs could have side effects with long-term use and pose important challenges to veterinary practitioners when dealing with OA [15]. The lack of effectiveness of tramadol for controlling joint function and pain in canine OA was recently shown [16]. There is a growing body of evidence that nutrients alter inflammatory responses and can therefore make a decisive contribution to the management of OA. Indeed, several nutritional substances have been tested so far in veterinary OA studies, such as omega 3 fatty acids and green mussel glycosaminoglycans. Some of these studies have provided interesting results, although mainly at the histological and molecular level [17,18]. Among the most used nutritional substances, the so-called classical chondroprotectors (e.g., chondroitin sulfate and glucosamine) have obtained heterogeneous results in veterinary studies, having proved effective by some investigators [17] and without benefit against OA pain by others [19,20]. A substantial need remains for safe and effective therapies that concurrently target chondrodegeneration, inflammation, and most importantly OA pain.

Recently, the endocannabinoid system is gaining increasing interest in veterinary medicine, particularly in the field of OA [21]. In this context, one of the most studied endocannabinoid-like substances is palmitoyl-ethanolamide (PEA) [22], an endogenous as well as foodborne compound, emerging as a promising dietary approach to inflammatory and/or painful disorders of the dog and cat [23]. PEA is the parent molecule of ALIAmides, i.e., a family of fatty acid amides acting through the so-called “autacoid local injury antagonism” (i.e., the ALIA mechanism) aimed at downmodulating hyperreactive cells (e.g. mast cells, microglia) [23]. Locally produced PEA has long been known to participate in the intrinsic control of pain [24] and to exert a tonic inhibitory control over the induction of nociceptive responses [25]. Its dietary supplementation was recently proved to relieve pain in natural [26,27,28] and experimental OA [29]. Palmitoyl-glucosamine (PGA)—the amide of palmitic acid and glucosamine—is an ALIAmide particularly suitable for orthopedic use [30] and it is listed in the EU-Catalogue of feed materials used in animal nutrition. Similarly to PEA [22,31], PGA has proven to downmodulate the release of bioactive mediators from hyperactive mast cells [32] and counteract OA-related joint mast cell hyperplasia [30]. Moreover, PGA exerted a significant pain relieving effect in a murine model of OA [33]. Using the same model, we have recently shown that micronized PGA exerts a superior activity to PGA on behavioral and locomotor deficits as well as histologic and radiographic join damage, confirming that particle size reduction effectively enhances the activity of PGA [30].

Curcumin is the main active ingredient in *Curcuma longa* L., commonly known as turmeric. It is a polyphenol with anti-inflammatory and anti-oxidant activities [34,35]. Because of low water solubility and chemical instability, the therapeutic usefulness of curcumin has been somewhat limited [36,37]. To increase the bioavailability of curcumin, a novel micronized and PGA complexed formulation has been developed (PGA-Cur). This formulation has recently proved to benefit OA dogs in a real-life survey, i.e., an observational study measuring the effectiveness of PGA-Cur in routine circumstances of everyday practice [38].

With all that said, the aim of the present study was to investigate whether the dietary supplementation with the ALIAmide PGA co-micronized with curcumin concurrently targets the main processes of OA (i.e., inflammation, chondrodegeneration, and sensitization) and helps in relieving pain and increasing mobility accordingly.

## 2. Materials and Methods 

### 2.1. Animals

This study was performed on Sprague-Dawley male rats (200–230 g, 7 weeks old, Envigo RMS Srl, S. Pietro al Natisone, Udine, Italy). Food and water were available ad libitum. The University of Messina Review Board for the care of animals authorized the study. Animal care was in accordance with Italian regulations on protection of animals used for experimental and other scientific purposes (D.M.116192) as well as with EEC regulations (O.J. of E.C. L 358/1 12/18/1986), and in compliance with the requirements of Italian Legislative Decree no. 26/2014 and subsequent guidelines issued by the Italian Ministry of Health on March 16, 2015; approval number (500/2018-PR) of 07/02/2018.

### 2.2. Reagents

Micronized PGA, curcumin, and co-micronized PGA-Cur were kindly provided by Epitech Group SpA (Saccolongo, Italy). Co-micronized PGA-Cur is the result of the joint ultra-micronization—by jet-milling technology—of a mixture made of PGA and curcumin in a 2:1 ratio by mass. The resulting particle size is in the range of 0.6 to 10 µm. All other compounds were obtained from Sigma-Aldrich, Milan, Italy. All chemicals were of the highest commercial grade available. All stock solutions were prepared in non-pyrogenic saline (0.9% NaCl, Baxter International, Rome, Italy).

### 2.3. Carrageenan-Induced Inflammation and Treatment Administration

Carrageenan (CAR)-induced inflammation is a recognized and highly reproducible model of acute inflammation and inflammatory pain [39,40]. Briefly, rats were anesthetized with 5.0% isoflurane in 100% O2 (Baxter International, Rome, Italy) and received a subplantar injection of carrageenan (CAR, 0.1 ml/rat of a 1% suspension in saline) (Sigma-Aldrich, Milan, Italy) with a 27-gauge needle into the right hind paw, as previously described [40,41]. Every compound was dissolved in carboxymethylcellulose (1% *w/v* in NaCl solution) and rats were randomly allocated to one of four groups and treated with

CAR + curcumin (10 mg/kg)
CAR + micronized PGA (20 mg/kg)
CAR + co-micronized PGA-Cur (2:1) (30 mg/kg)
CAR + carboxymethylcellulose (vehicle group)

As a sham group, saline was injected instead of CAR (control group). Each study compound (or vehicle) was administered orally by gavage as a single administration 30 min before CAR injection (N = 6 animals/group). Doses were chosen based on a dose–response study carried out in our lab and previous results [30]. The animals were sacrificed at 6h post CAR injection by isoflurane overdose. All analyses were performed in a blinded manner (i.e., the operator who assessed the below detailed parameters and the statistician who analyzed the data were both unaware of the treatment group).

#### 2.3.1. Assessment of Paw Edema

Paw volume (mL) was measured using a plethysmometer (Ugo Basile, Varese, Italy) immediately prior to CAR injection and thereafter at 30 min and hourly intervals for 6 h. Edema was expressed as the increase of paw volume at each time point relative to pre-injection value [30]. Results are reported as paw-volume change (mL).

#### 2.3.2. Assessment of Inflammatory Pain

The hyperalgesic response to heat was determined at different time points (0, 30 min, and hourly intervals for 6 h) based on the method described by Hargreaves et al. [42], using a Basile Plantar Test (Ugo Basile, plantar test apparatus 7371; power requirement: 230–115 V, 60–50 Hz, 0.6 A maximum) as previously described [41]. Results are expressed as paw withdrawal latency changes (seconds).

#### 2.3.3. Histopathological Analysis of Paw Tissue

Histological analysis of hematoxylin and eosin (H/E)-stained paw tissue was performed as previously described [41]. Briefly, the degree of tissue inflammation was evaluated on a 6-point score, from 0 (no inflammation) to 5 (severe inflammation) [43]. The photographs obtained (N = 5 photos from five slides for each sample) were collected from all animals in each experimental group. The histological coloration (five slides for each same sample) was repeated three times on different days.

#### 2.3.4. Evaluation of Myeloperoxidase Activity

Myeloperoxidase (MPO) activity, an index of neutrophilic granulocyte infiltration, was evaluated as previously described [30,41]. Briefly, the rate of change of the absorbance was measured spectrophotometrically at 650 nm. MPO activity was measured as the quantity of enzyme degrading 1 mM of peroxide/min at 37 °C and expressed as units per gram of wet tissue weight.

### 2.4. Monoiodoacetate-Induced Osteoarthritis and Treatment Administration

The intra-articular injection of sodium monoiodoacetate (MIA) is a technically straightforward model that closely mimics the behavioral and pathological features associated to OA [44,45]. In particular, it is recognized as a standard for modeling joint pain [44,45,46,47]. Similarly to natural OA, MIA-induced joint pain progressively develops the characteristics of neuropathic pain [45,48], whose prominent symptom is allodynia [49,50]. Intra-articular injection of MIA also results in cartilage degeneration as well as joint inflammation [44].

Briefly, rats were anesthetized with 5.0% isoflurane in 100% O2 and a volume of 25 µl saline + 3 mg of MIA was injected into the right knee joint using a 50 μl Hamilton syringe with a 27 gauge needle [41,51]. The left knee received an equal volume of saline. As a sham group, saline was injected instead of MIA (control group). Each study compound (or vehicle) was administered orally by gavage as a repeated administration three times per week for 21 days, starting the third day after MIA injection (N = 10 animals/group). Dissolution vehicle and doses were the same as CAR. On day 21 post-MIA injection, rats were sacrificed by anesthetic overdose and perfused with 4% paraformaldehyde. All analyses were performed in a blinded manner as detailed above.

#### 2.4.1. Assessment of Mechanical Allodynia

Mechanical allodynia was evaluated using a dynamic plantar Von Frey hair esthesiometer on day 0 and 3, 7, 14, and 21 days post-injection (Ugo Basile, Comerio, Italy) as previously described [30,41]. Briefly, the Von Frey-type 0.5 mm filament began to move below the metatarsal region with a gradually increasing force until the rat removed its paw. The force required to produce a paw withdrawal reflex (i.e., the paw withdrawal threshold (PWT)) and the time interval between the stimulus and the response (i.e., paw withdrawal latency (PWL)) were automatically detected and recorded (in grams and seconds, respectively). Given the features of the model (MIA-induced knee OA) and the evocation of pain (application of the non-noxious von Frey hairs to the paw) this technique is measuring referred pain or secondary allodynia [52].

#### 2.4.2. Motor Function Analysis 

Motor functional recovery of the rear limb was evaluated by walking track analysis. This is a reliable and easily quantifiable noninvasive method based on gait analysis by means of specific footprint parameters (e.g., stride length) obtained by wetting the rat’s hind feet with water soluble black ink and allowing it to walk down a track covered with strips of white paper [41]. As previously described [30], the footprint measures were combined to yield the SFI (sciatic functional index), whose values range from −100 (total impairment) to 0 (normal function). SFI values in the control group were assumed as zero. The analysis was performed before MIA injection and on a weekly basis (at 7, 14, and 21 days post-injection). The percentage of motor function recovery was calculated at each time point by subtracting the mean value registered in the treated group to the corresponding mean value of the vehicle group, using the following formula,

$$\mathrm{Motor}\;\mathrm{function}\;\mathrm{recovery}\;(\%)=\frac{\mathrm{SFI}\;(\mathrm{vg})-\mathrm{SFI}\;(\mathrm{tg})}{\mathrm{SFI}\;(\mathrm{vg})}\times 100$$

with (vg) = vehicle group and (tg) = treated group.

#### 2.4.3. Histological Analysis of Tibiofemoral Joint Cartilage Damage

The tibiofemoral joints were dissected immediately after sacrifice and post-fixed in neutral buffered formalin (containing 4% formaldehyde) as previously described [41]. Mid-coronal tissue sections (5 μm) were stained with H/E and observed by light microscopy [30]. A modified Mankin histologic scoring system was used to evaluate cartilage damage, from 0 (normal histology) to 12 (complete disorganization and hypocellularity) [49]. The photographs obtained (N = 5 photos from five slides for each sample) were taken from all animals in each experimental group. The histological coloration (five slides for each same sample) was repeated three times on different days.

#### 2.4.4. Measurement of Pro-Inflammatory, Sensitizing, and Matrix Degradation Serum Markers 

On day 21 post-MIA injection, rats were sacrificed and serum were taken and stored at −80 °C. Subsequently, the concentration of tumor necrosis factor alpha (TNF-α), interleukin-1beta (IL1-β), nerve growth factor (NGF), and matrix metalloproteinase-1-3-9 (MMP1, MMP3, MMP9) were measured in serum using commercial colorimetric ELISA kits (TNF-α, IL-1β, and NGF: Thermo Fisher Scientific, DBA s.r.l. Milan Italy; MMP-1, MMP- 3, and MMP-9: Cusabio, DBA s.r.l. Milan Italy).

### 2.5. Data Analysis

Data are expressed as mean ± standard error of the mean (SEM) of N observations (N = number of animals analyzed) with the exception of the ordinal level variable (i.e., 6-point histological score), for which median and range were used. The latter were analyzed by Kruskal–Wallis test followed by Dunn’s test for post hoc comparisons with Bonferroni–Holm p correction. Histopathological figures are representative of at least three independent experiments performed on different days. In all other experiments, one- or two-way ANOVA followed by a Bonferroni–Holm post hoc test for multiple comparisons were used. Data were analyzed using SAS v9.2 (SAS Institute, Cary, NC, USA). The significance threshold was set at 0.05. Exact p values are reported, unless less than 1 out of 10,000 (reported as *p* < 0.0001), 0.0001 being the lower limit for the statistical program.

## 3. Results

### 3.1. Effect on Carrageenan-Induced Paw Edema

**Figure 1:**
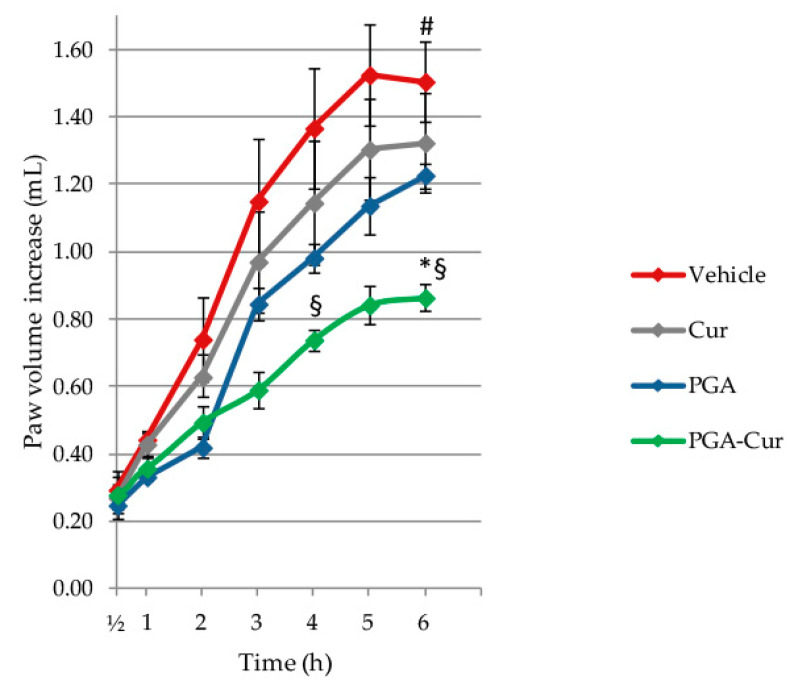
Anti-inflammatory effect of palmitoyl-glucosamine (PGA) and curcumin (Cur) given individually or in a co-micronized formulation (PGA-cur) on carrageenan-induced paw edema. Inflammation was assessed as paw volume increase (mL) after CAR injection relative to pre-injection value (i.e., basal condition). Results are expressed as means ± standard error of the mean (SEM) (N = 6 animals/group). PGA = micronized palmitoyl-glucosamine. # *p* < 0.0001 vs. basal condition; * *p* < 0.05 vs. vehicle; § *p* < 0.05 vs. PGA.

CAR resulted in a steady increase of paw volume after injection, with the edema being significantly increased at 6 h compared to basal condition (*p* < 0.0001). Co-micronized PGA-Cur significantly reduced paw edema at 6h (*p* = 0.0410), while the single compounds (i.e., curcumin or PGA alone) did not reach significant effect at any time point (Figure 1).

### 3.2. Effect on Carrageenan-Induced Inflammatory Pain

CAR-induced hyperalgesia was expressed as paw withdrawal latency changes (seconds), i.e. the less the latency the more severe the hyperalgesia (i.e., inflammatory pain). As summarized in Table 1, paw withdrawal latency significantly decreased over time after CAR injection (from 14.5 ± 0.07 to 5.6 ± 0.22 s); this was counteracted by PGA starting at the fifth hour (*p* = 0.0027). Conversely, the effect of co-micronized PGA-Cur appeared at earlier time points (i.e., starting from the first h after CAR injection, *p* = 0.0045) and was significantly greater compared to the compounds given individually.

### 3.3. Effect on Carrageenan-Induced Histological Inflammation and Neutrophil Infiltration into the Paw

**Figure 2:**
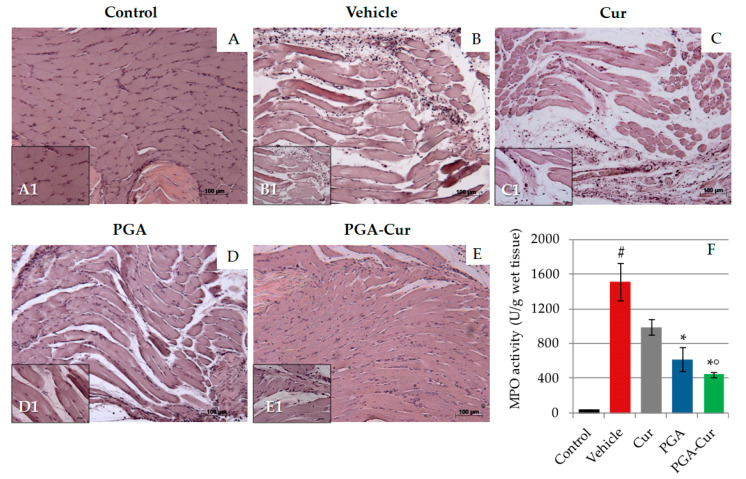
Anti-inflammatory effect of the study compounds in CAR-injected paw. Representative images of H/E-stained paw sections (**A–E**). Intraplantar injection of CAR-induced marked edema and cellular infiltration (**B**) compared to control animals (**A**). Histopathological changes appeared not to benefit from a single dose of curcumin (**C**), while PGA administration counteracted both tissue edema and cellular infiltration (**D**). The co-micronized formulation (single administration, 30 min prior to CAR) prevented almost completely the inflammatory changes (**E**). The histological findings mirrored the neutrophil infiltration into the paw as measured by myeloperoxidase (MPO) activity (**F**). Values are means ± standard error of the mean of 6 animals for each group. Cur = curcumin; PGA = micronized palmitoyl-glucosamine; PGA-Cur = co-micronized palmitoyl-glucosamine and curcumin. # *p* < 0.05 vs. control; * *p* < 0.05 vs. vehicle; ° *p* < 0.05 vs. Cur. The p values are reported in the text.

CAR-injected paws showed marked edema as well as pronounced inflammatory cell infiltration (Figure 2B) compared to control animals (Figure 2A). Treatment with curcumin did not appear to exert any anti-inflammatory effect (Figure 2C). PGA only modestly reduced inflammatory cell infiltration (Figure 2D), whereas the co-micronized PGA-Cur formulation counteracted CAR-induced inflammatory changes into the injected paw (Figure 2E). Accordingly, the severity of histological inflammatory score was significantly decreased by oral administration of PGA-Cur, but not by the compounds given individually at the presently used dose (Table 2).

A great and statistically significant infiltration of neutrophils was observed in the injected paw (*p* = 0.0060; Figure 2F). Both PGA and its co-micronized formulation with curcumin significantly inhibited cell recruitment (*p* = 0.0300 and *p* = 0.0202, respectively), while the effect of curcumin given individually did not reach statistical significance (Figure 2).

### 3.4. Effect on Monoiodoacetate-Induced Neuropathic Pain

**Figure 3:**
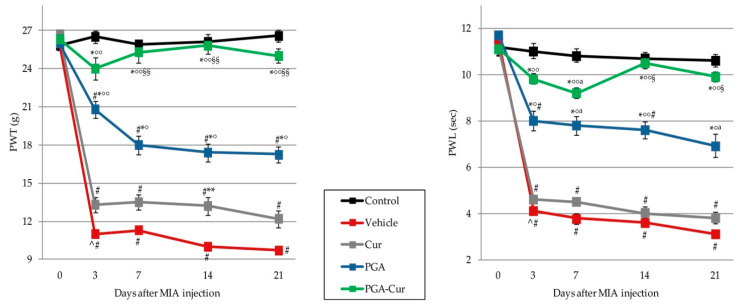
Effect of PGA and curcumin (Cur) given individually or in a co-micronized formulation (PGA-Cur) on monoiodoacetate-induced neuropathic pain. The effect was assessed on secondary allodynia by measuring paw withdrawal threshold (PTW) and paw withdrawal latency (PWL) before and after 3, 7, 14, and 21 days from MIA knee injection. Data are expressed as mean ± standard error of the mean (N = 10/group). PGA = micronized palmitoyl-glucosamine. ^ *p* < 0.0001 vs. previous time point; # *p* < 0.0001 and ^a^ *p* < 0.05 vs. control; * *p* < 0.0001 and ** *p* = 0.0295 vs. vehicle; °*p* < 0.05 and °° *p* < 0.0001 vs. Cur; §§ *p* < 0.0001, § *p* < 0.005 vs. PGA.

MIA injection led to secondary allodynia from day 3 onward, as indicated by the significant drop of PWT and PWL in the vehicle group (*p* < 0.0001 for all comparisons; Figure 3). The effect of curcumin was significant limited to PWT and only at day 14 (*p* = 0.0295). On the contrary, dietary supplementation with PGA (and even more so PGA-Cur) reverted allodynia at either threshold or latency level (Figure 3). The effect was prompt (day 3), statistically significant and superior to that of curcumin at all time points (Figure 3). In particular, the co-micronized formulation completely normalized both the threshold and latency time after only 3 days of administration, as the values registered in the treated group did not differ from the control one.

### 3.5. Effect on Monoiodoacetate-Induced Locomotor Dysfunction

**Figure 4:**
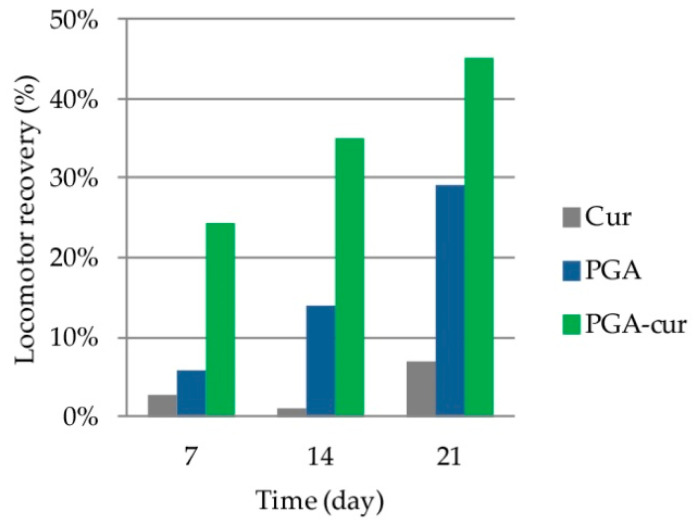
The effect of palmitoyl-glucosamine (PGA) and curcumin (Cur) given individually or in a co-micronized formulation (PGA-Cur) on locomotor recovery.

Walking track analysis was here used to calculate the motor function changes due to MIA injection and the investigated oral supplements. As expected, MIA injection time-dependently increased the locomotor deficit with the mean SFI value reaching −56.5 ± 1.30 at day 21 (*p* < 0.0001 versus the control group; Table 3). Oral administration of curcumin did not reach any significant effect compared to vehicle, while PGA resulted in a significant motor function recovery after three week-supplementation period (29%; *p* = 0.0003). PGA-Cur yielded an earlier and superior effect, resulting in 35% and 45% locomotor recovery compared to vehicle, at day 14 (*p* = 0.0017) and day 21 (*p* < 0.0001), respectively (Figure 4).

### 3.6. Effect on Monoiodoacetate-Induced Joint Cartilage Damage

**Figure 5:**
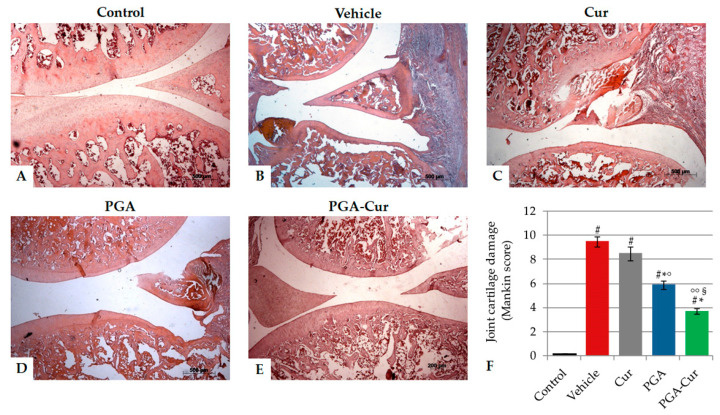
Protective effect of the study compounds on monoiodoacetate -induced histological damage to the knee joint cartilage. Representative images of hematoxylin and eosin (H&E)-stained sections in the sagittal plane. Tibiofemoral joints injected with MIA showed erosion and roughening of the articular cartilage, microfractures in the fibrocartilage, as well as expansion of the synovial membrane (**B**) compared to control joints (**A**). Curcumin 10 mg/kg (**C**) and PGA 20 mg/kg (**D**) administered alone exerted a little protective effect against joint damage. The oral administration of PGA co-ultramicronized with curcumin (PGA-Cur, 30 mg/kg) counteracted the histological damage to a higher extent (**E**), as also shown by the greater and significantly higher decrease in the severity score of the osteochondral damage (i.e., modified Mankin score, F). Values are means ± standard error of the mean of 10 animals for each group. See text for further details. # *p* < 0.0001 vs. control; * *p* < 0.0001 vs. vehicle; ° *p* < 0.05 and °° *p* < 0.0001 vs. Cur; § *p* < 0.0001 vs. PGA.

Histological changes were microscopically assessed in H/E-stained sections to detect MIA-induced cartilage degeneration of the tibiofemoral joints. A general thinning of joint cartilage, with loss of the superficial zone and denudation in the deep zone as well as erosions and surface roughening, was evident in the vehicle-treated group (Figure 5B) compared to controls (Figure 5A). When administered alone, PGA and curcumin showed a minimal protective effect against chondrodegeneration (Figure 5C,D). On the contrary, supplementation with the co-micronized formulation (PGA-Cur) exerted an important protection against histological damage (Figure 5E).

MIA injection resulted in a statistically significant increase of the mean histologic score, which was counteracted by PGA but not curcumin by itself (Figure 5F). Three-week oral administration of the co-micronized formulation had a significantly greater protective effect against cartilage damage compared to PGA alone (Figure 5F).

### 3.7. Effect on Monoiodoacetate-Induced Increase of Proinflammatory, Sensitizing, and Matrix Degradation Mediators

**Figure 6:**
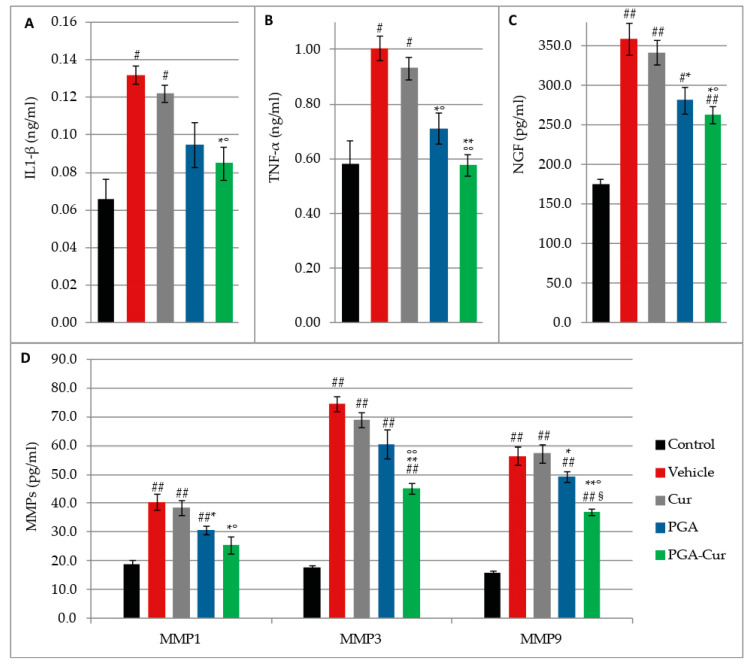
Effect of PGA and curcumin (Cur) given individually or in a co-micronized formulation (PGA-Cur) on monoiodoacetate-induced increase in the serum level of cytokines (IL1-β, TNF-α), nerve growth factor (NGF), and metalloproteases (MMP1, 3, 9). Data are expressed as mean ± standard error of the mean (N = 10/group). # *p* < 0.05 and ## *p* < 0.0001 vs. control; *p < 0.05 and **p < 0.0001 vs. vehicle; °*p* < 0.05 and °°*p* < 0.0001 vs. Cur; § *p* < 0.0001 vs. PGA.

MIA injection resulted in a significant 50% and 42% increase of IL1-β and level, respectively (Figure 6A,B). Furthermore, NGF concentration doubled in response to MIA injection (Figure 6C) and the percentage increase of MMP level was even higher (Figure 6D). Curcumin did not show any significant effect on MIA-induced increase (Figure 6A–D), while PGA significantly reduced the level of TNF-α (*p* = 0.0060), NGF (*p* = 0.0359), MMP1 (*p* = 0.0288), and MMP9 (*p* = 0.0175) compared to vehicle (Figure 6B–D). PGA-Cur significantly counteracted MIA-induced increase of all the investigated mediators (Figure 6A–D). In particular, its oral administration completely normalized IL1-β, TNF-α, and MMP1, whose levels did not differ from vehicle treated group (Figure 6A,B,D). Besides being superior to curcumin on all the investigated mediators, the effect of the co-micronized formulation also resulted significantly greater compared to PGA on the MIA-induced increase of MMP9 (*p* = 0.0003).

## 4. Discussion 

This is the first time that oral supplementation with the novel composite made from co-micronizing PGA and curcumin (PGA-Cur 2:1 ratio, 30 mg/kg) has been investigated in two well-established models of inflammatory and OA pain, respectively. The preclinical animal models were chosen because they offer great potential to understand OA pathophysiology and study new interventions [44,45,52], while at the same time they allow to overcome issues related to the etiological and clinical heterogeneity of the naturally occurring canine OA.

Our findings point out the benefits resulting from supplementing PGA-Cur in terms of relieving inflammation, hyperalgesia, and secondary allodynia, the latter being well-known hallmarks of neuropathic pain. As low-grade chronic inflammation and mixed pain (i.e., the co-occurrence of inflammatory and neuropathic pain) are well recognized mechanisms of OA [11], PGA-Cur emerges as a possible mechanism-based dietary approach to OA. The observation is in agreement with the results of a recently published survey on 181 dogs with OA daily supplemented with PGA-Cur (20 mg/kg b.w.) for a two month-period [38]. A significant decrease of veterinary assessed lameness score and a parallel relief of pain on joint palpation/manipulation were found [38]. Owner-assessed pain behavior similarly decreased as early as the first month of dietary supplementation and an overall locomotor recovery rate of 44% was recorded [38].

In a previous study evaluating the 30 mg/kg dose of PGA (either in the naïve or micronized formulation) it was found that micronization yielded superior and earlier results compared to naïve formulation [30]. At the 20 mg/kg dose investigated in the present study, micronized PGA failed to be effective on some parameters. For instance, CAR-induced paw edema (Figure 1) and tissue damage (Table 2) as well as MIA-induced increase in IL1-β and MMP3 content did not show any significant benefit from micronized PGA (Figure 6A,D). This might imply that higher doses are needed if micronized PGA is used alone, unless it is co-micronized with curcumin. Together with the apparent lack of efficacy of curcumin (10 mg/kg dose) that emerged from the present study, this suggests a synergistic effect between PGA and curcumin at the doses tested here. In these respect, it is worth mentioning that the effect of curcumin administered as a sole treatment in the MIA model are observed for much higher doses compared to the one used here (i.e., tenfold and higher) [53].

CAR-induced primary hyperalgesia (i.e., a measure of inflammatory pain) was reduced by a single dose PGA-Cur orally administered 30 minutes before injection. The effect was statistically significant as early as the first post injection hour and reached an extent of 48% decrease at the sixth post-injection hour, when pain was highest (Table 1). More importantly, the pain relieving effect of PGA-Cur was superior to micronized PGA or curcumin administered alone. Again, a synergistic effect of the co-micronized compounds might explain the result. 

Dietary supplementation with PGA-Cur reverted MIA-induced secondary allodynia at either threshold or latency level (Figure 3), thus suggesting an effect on peripheral sensitization. This was also confirmed by the effect of PGA-Cur in counteracting MIA-induced increase of NGF serum levels (Figure 6C). NGF is significantly increased in dogs with chronic lameness [54] and plays a key role in nociceptor sensitization after tissue injury, and reducing NGF levels is currently considered one of the most promising approach to modulating OA pain [11,55]. Further mediators are currently recognized as nociceptor sensitizers, e.g., IL1-β, TNF-α, and MMPs [48]. They are all considered to contribute to natural and experimentally-induced OA pain, either indirectly through promoting low-grade chronic inflammation and cartilage degradation, or directly through sensitization of peripheral afferents [11,48,56,57,58]. In particular, joint and serum MMPs are associated with pain progression in the MIA model [56]. We found that MIA-induced increase in serum levels of TNF-α and MMP1 was completely abolished by repeated administration of PGA-Cur (three times per week from the third day after MIA injection; Figure 6B,D). The pain relieving effect of PGA-Cur might thus depend—at least in part—on the decrease of proinflammatory and sensitizing mediators.

Although the present study did not directly address the possible mechanism(s) of action sustaining the benefit of PGA-Cur, some hypotheses might be drawn. As previously suggested, synergism between the ALIAmide PGA and curcumin is one of the possible explanations. Actually, several evidences support the use of curcumin in OA, mainly based on antioxidant in vitro property and a few clinical studies in humans and dogs, all performed with highly available formulations [37,59,60,61]. Most notably, PGA—especially in the micronized form—not only relieves inflammation and pain [23,30,33], but also exerts an entourage effect on endocannabinoids and related ALIAmides (e.g., PEA), as suggested by some preliminary observations [62]. The endocannabinoid system is now emerging as a pivotal player in joint functioning and its alterations contribute to OA pathology [48,63,64]. In the knee of MIA-injected animals, a tremendous increase of cannabinoid receptors (e.g., CB1 and CB2) and endocannabinoid-degrading enzymes (amide hydrolases) was recently found [56]. Changes of the endocannabinoid system—which is a well-described feature of human and canine OA [21,65]—do not only occur at the joint level, but also at the spinal level [48,66]. Inhibition of endocannabinoid degradative enzymes and endocannabinoid receptor agonism are two strategies found to reduce joint inflammation and pain in different arthritis and osteoarthritis animal models [48,67,68,69]. The issue has been extensively reviewed in [70]. Activation of the endocannabinoid system is thus currently viewed as a novel strategy to reduce OA symptoms and correspondingly increase quality of life [63]. In this framework, the entourage effect of PGA on endocannabinoids and endocannabinoid-related compounds [62] might well explain the results obtained in the present study.

## 5. Conclusions

Chronic pain (first and foremost OA pain) is presently underdiagnosed and inadequately managed in non-verbal patients, like dogs and cats, and can result in premature euthanasia [7,71]. An important endogenous pain modulation impairment has recently been shown in dogs with spontaneous OA and considered to significantly contribute to chronic pain [72]. Endogenous ALIAmides—and most particularly PEA—are involved in inhibitory pain modulation [24,25]. A co-micronized formulation of the ALIAmide PGA with the antioxidant curcumin (PGA-Cur) was here found to relieve inflammatory as well as neuropathic pain and to successfully address locomotor deficits, inflammatory processes and joint structural degradation during experimentally induced OA. These data are clinically confirmed by preliminary findings from a real-life survey in dogs with OA [38]. PGA-Cur is safe, with the oral LD₅₀ of both components (i.e., curcumin and PGA) being greater than 2000 mg/kg body weight [30,73]. With all that said and although well-designed clinical studies are needed PGA-Cur emerges as a useful dietary intervention in the multimodal management of canine and feline OA.

## Figures and Tables

**Table 1:** The effect of PGA and curcumin (Cur) given individually or in a co-micronized formulation (PGA-Cur) on inflammatory pain measured as thermal hyperalgesia. Data (mean and standard error of the mean (SEM), N = 6/group) are expressed as paw withdrawal latency change (in seconds) at the consecutive time points. The less was the latency the more severe the hyperalgesia.

	Time (h)	0	1/2	1	2	3	4	5	6
Vehicle	Mean	14.5	14.2	10.5 ^##^	7.9 ^#^	7.6	6.8	5.9 ^#^	5.6
	SEM	0.07	0.13	0.06	0.11	0.12	0.21	0.24	0.22
Cur	Mean	14.5	14.2	10.6	8.1	7.8	7.0	5.9	5.7
	SEM	0.10	0.04	0.05	0.21	0.04	0.10	0.14	0.12
PGA	Mean	14.2	14.1	10.0	7.7	7.4	7.4	8.0 *	8.1 *
	SEM	0.07	0.06	0.06	0.09	0.11	0.10	0.20	0.18
PGA-Cur	Mean	14.2	14.4	11.4 *°^§^	9.6 *°^§^	9.5 *°^§^	9.3 *°^§^	8.5 *°	8.3 *°
	SEM	0.07	0.09	0.12	0.04	0.04	0.08	0.21	0.24

PGA = micronized palmitoyl-glucosamine. ## *p* < 0.0001; # *p* < 0.005 vs. previous time point; * *p* < 0.005 vs. vehicle; § *p* < 0.05 vs. PGA; ° *p* < 0.05 vs. Cur.

**Table 2:** The effect of PGA and curcumin (Cur) given individually or in a co-micronized formulation (PGA-Cur) on the severity of the histological inflammatory score in carrageenan-injected paw. The score was a 6-point scale, from 0 (no inflammation) to 5 (severe inflammation) [43].

	Control	Vehicle	Cur	PGA	PGA-Cur
N	6	6	6	6	6
Min	0	4	3	2	2
Median	0	4 ^#^	3.5	3	2*
Max	0	5	4	4	3
Mean	0	4.2	3.5	3.0	2.2
SEM	0	0.2	0.2	0.4	0.2

PGA = micronized palmitoyl-glucosamine. SEM = standard error of the mean. # *p* < 0.0001 vs. control; * *p* < 0.05 vs. vehicle.

**Table 3:** The effect of the study compounds on locomotor deficit, measured as SFI (ranging from −100 = total impairment to 0 = normal function, the latter being assumed in the control group). Values are expressed as mean ± standard error of the mean (N = 10/group).

	Vehicle	Cur	PGA	PGA-Cur
Day 7	−35 ± 2.11 ^#^	−34 ± 2.21	−33 ± 2.26	−26.5 ± 1.83
Day 14	−47 ± 2.38 ^#^	−46.5 ± 2.48	−40.5 ± 2.63	−30.5 ± 1.89 *°
Day 21	−56.5 ± 1.30 ^#^	−52.5 ± 1.86	−40 ± 2.11 *°	−31 ± 2.33 **°°

Cur = curcumin; PGA = micronized palmitoyl-glucosamine; PGA-Cur = co-micronized palmitoyl-glucosamine and curcumin. # *p* < 0.0001 vs. control; * *p* < 0.05 and ** *p* < 0.0001 vs. vehicle; ° *p* < 0.05 and °° *p* < 0.0001 vs. Cur.
